# Nutritive Material

**Published:** 1896-06

**Authors:** 


A QUART of milk, three-quarters of a pound of moderately fat beef—sirloin steak, for instance—and five ounces of wheat flour, all contain about the same amount of nutritive material; but we pay different prices for them, and they have different values for nutriment. The milk comes nearest to being a perfect food. It contains all of the different kinds of nutritive materials that the body needs. Bread made from wheat flour will support life. It contains all the necessary ingredients for nourishment, but not in proportions best adapted for ordinary use. A man might live on beef alone, but it would be a very one-sided and imperfect diet. But meat and bread together make the essentials of a healthful diet. Such are the facts of experience. The advancing science of later years explains them. This explanation takes into account, not simply quantities of meat and bread and milk and other materials which we eat, but also the nutritive ingredients or "nutrients" which they contain.—*Sanitarian.*

